# Photosynthetic Traits Underlying Yield Differences in Irrigated and Rainfed Soybean Systems in the Mississippi Delta

**DOI:** 10.1002/pei3.70178

**Published:** 2026-07-01

**Authors:** Srinivasa R. Pinnamaneni, Isabel M. Lima, Jean A. Beacorn

**Affiliations:** ^1^ Western Colorado Research Center‐Grand Valley, Colorado State University Fruita Colorado USA; ^2^ USDA‐ARS, Southern Regional Research Center, Commodity Utilization Research New Orleans Louisiana USA

**Keywords:** instantaneous carboxylation efficiency, instantaneous water use efficiency, moisture deficit stress, photosynthesis, stomatal conductance, transpiration rate

## Abstract

Understanding photosynthetic processes under field conditions is critical for identifying physiological determinants of seed yield (SY) in soybean. This study evaluated leaf gas exchange and chlorophyll fluorescence traits in seven soybean cultivars grown under irrigated (IR) and rainfed (RF) environments in the Mississippi Delta. At the beginning seed stage (R5), key physiological parameters—including net photosynthetic rate (A), stomatal conductance (gs), intercellular CO_2_ concentration (Ci), transpiration rate (E), instantaneous water‐use efficiency (WUE = A/E), instantaneous carboxylation efficiency (ICE = A/Ci), electron transport rate (ETR), and photosystem II quantum yield (ΦPSII)—were measured. Seed yield was determined at physiological maturity (R8). Under irrigated conditions, A and ICE showed the strongest positive associations with SY, whereas under rainfed conditions, WUE emerged as the primary predictor of yield. Cultivars DS 25‐1, Dyna‐Gro 4516×, LG03‐4561‐14, and P37A78 exhibited consistently superior physiological performance across both water regimes. These findings highlight the importance of integrating gas exchange and fluorescence‐based traits into trait‐based selection strategies. However, given the limited number of cultivars, location, and growing seasons evaluated, these results should be interpreted as indicative rather than definitive. This study provides one of the first field‐based comparisons of photosynthetic traits across maturity groups III–V within the Early Soybean Production System, offering insights into physiological indicators of yield stability under contrasting water regimes.

## 1 Introduction

Soybean ( *Glycine max* (L.) Merr.) production economics worldwide are significantly affected by periodic abiotic stresses such as drought, flood, high temperatures, and biotic stresses such as anthracnose, bacterial blight, and charcoal rot diseases. In the lower Mississippi Delta (LMD) region, USA, both drought stress and intermittent flooding are major constraints limiting soybean productivity and yield stability, with studies reporting yield declines between 24% and 50% (Frederick et al. 2001). The LMD, characterized by a humid climate, is among the most productive agricultural regions in the United States; however, rainfall distribution is often uneven (Kottek et al. 2006). As a result, irrigating when soil water does not meet crop water demand is necessary for optimizing production (Anapalli et al. 2019).

Soil moisture‐deficit conditions can affect photosynthesis and the physiological performance of soybean genotypes grown under varied soil‐water‐crop management practices (Cornic 2000). Since the photosynthetic mechanism involves several components such as photosynthetic pigments and photosystems, electron transport system, and CO_2_ reduction pathways, any damage at any level caused by stress may reduce the overall carbon assimilation capacity (Ashraf and Harris 2013; Ohashi et al. 2006; Roche 2015). Hence, reduced CO_2_ assimilation is critical since it can lead to total crop losses or reduced grain yields (Cheng et al. 2018; Cotrim et al. 2021). Gas exchange analysis is an important tool in plant breeding for identifying cultivars with improved tolerance to adverse environmental conditions (Roche 2015; Urban et al. 2017). Reduced root water uptake due to water deficit in the soil can significantly reduce cell turgor and expansion growth resulting in reduced leaf area available for light harvest for photosynthesis, canopy conductance for CO_2_ intake, and increased canopy temperature leading to a decline in photosynthetic carbon assimilation rates (Jumrani and Bhatia 2019). Reduction in photosynthetic traits under environmental stresses could be attributed to many factors, such as a decline in leaf area, premature leaf senescence, reduced photosynthetic rates, impaired electron transport, oxidation of chloroplast lipids, poor ATP (adenosine triphosphate), and NADPH (nicotinamide adenine dinucleotide phosphate) synthesis (Gilbert et al. 2011; Roche 2015).

Further, environmental stress‐induced stomatal closure, modifications in light‐harvesting systems and proteins, carbon reduction cycle in the chloroplast and transport out of the Calvin‐Benson cycle, and utilization of photosynthates may lead to reduced CO_2_ assimilation (Ohashi et al. 2006; Roche 2015; Vongcharoen et al. 2019). By using chlorophyll fluorescence phenotyping and mixed‐model analysis, they found that tolerant genotypes with higher yield stability exhibited significantly higher Soil Plant Analysis Development (SPAD), non‐photochemical quenching (NPQt), and maximum quantum efficiency of PSII (FvP/FmP) under drought, along with reduced leaf thickness. Further, maintaining adequate soil water supplies for sustaining transpiration rate in irrigated soybean systems could be a viable strategy as it increases both WUE and seed yield (Tong et al. 2019). Genotypic differences in drought tolerance using photosynthetic and related physiological parameters have been reported earlier (Jumrani and Bhatia 2019).

They also identified superior genotypes suited for moisture deficit stress through a selection strategy involving canopy temperature, photosynthesis rate, specific leaf weight, and photosystem II efficiency. Another greenhouse study revealed differential physiological response of genotypes to contrasting moisture regimes, which was used to classify them for drought tolerance (Todeschini et al. 2019). In recent years, chlorophyll fluorescence measurements have been used as a powerful tool to understand and quantify photosynthetic efficiency not related to stomatal conductance (Kalaji and Guo 2008; Swoczyna et al. 2022). Chlorophyll fluorescence measurements help to understand changes in plant photochemistry from various biotic and abiotic sources in the plant and environment; as such, they can help in screening a large number of genotypes quickly for desirable photosynthetic traits (Cotrim et al. 2021; Jumrani and Bhatia 2019).

The highest quantum yield of photosystem II (ΦPSII), expressed as the ratio of variable fluorescence to maximal chlorophyll fluorescence (Fv/Fm), is a good indicator of photoinhibition in crop species under biotic as well as abiotic stresses. Many studies established the positive association of photosynthetic parameters for higher grain productivity such as higher flag leaf photosynthesis in wheat (Duvnjak et al. 2024); higher photosynthetic capacity, longer photosynthetic duration, and higher chlorophyll content in corn (Yan et al. 2021); and higher photosynthesis rate in rice (Xiong 2024). A meta‐analysis of 112 studies revealed that A, E, g_s_, Ci and WUE had significantly impacted the productivity under drought (Zhang et al. 2018) while fluorescence measurements along with gas exchange parameters, play a critical role in deciphering the physiological mechanisms associated with photosynthesis and productivity (Urban et al. 2017).

Crop cultivars and the practices for managing them can change with time, potentially affecting landscape water and energy balances. An earlier soybean production system in the Mississippi Delta consisted of planting maturity group (MG) VI and VII cultivars in May and June that matured in October and November and were prone to terminal moisture deficit stress that typically occurred between August and September. A relatively newer alternative to this system, known as the early soybean production system (ESPS), widely adopted today, was developed to overcome the terminal (late season) drought, which is likely to occur in this region's climate during August and September (Heatherly and Elmore 2004).

The ESPS primarily involves planting early maturing cultivars falling under MG III, IV, and V in April or May, which mature in August or September (Smith et al. 2019). Further, another study measured chlorophyll fluorescence parameters at R5 stage across cultivars and found that yield gains have been realized primarily through cultivar breeding, with extended reproductive periods as a key mechanism (Sun et al. 2025). Recent studies have highlighted how photosynthetic parameters such as WUE and ICE influence yield through interconnected physiological mechanisms. WUE is governed by stomatal and mesophyll conductance, with drought conditions prompting a trade‐off between WUE and nitrogen‐use efficiency—ultimately shaping biomass accumulation (Broeckx et al. 2014). While water stress and elevated CO_2_ increase WUE, yield outcomes depend on carbon allocation dynamics and stomatal acclimation, emphasizing the complexity of linking WUE directly to productivity (Gea‐Izquierdo et al. 2015; Živčák et al. 2008). Tools to estimate key photosynthetic parameters like electron transport rate and mesophyll conductance have enabled insights into photoprotective strategies and PSII efficiency essential for sustaining carbon assimilation and yield under stress (Moualeu‐Ngangue et al. 2017). While these studies have improved our understanding of how photosynthetic parameters such as WUE and ICE influence yield through mechanisms like carbon allocation and photoprotection, important gaps remain.

Specifically, field‐based gas exchange and fluorescence comparisons across MG III–V within ESPS under IR versus RF in the Mississippi Delta are limited. Soybean breeders could include better correlated photosynthetic parameters in their selection criteria to breed efficient stress tolerant lines. Hence, the objective of this study was to characterize photosynthetic parameters estimated from the gas exchange and chlorophyll fluorescence measurements on the SY of seven soybean cultivars grown under rainfed (RF) and irrigated (IR) environments in the humid climate of the MS Delta region. Further, the photosynthetic traits' association with SY was examined through correlation analysis. The seven cultivars included released and pre‐released lines bred for flood, drought, heat stress tolerances, charcoal rot resistance, and transgenic cultivars produced by seed companies for cultivation in the MS Delta.

## 2 Materials and Methods

Experiments were conducted at the USDA‐ARS, Crop Production Systems Research Unit farm located in Stoneville, MS, USA (33° 42′ N, 90° 55′ W, elevation: 32 m asl) on a Dundee silt loam (fine silty, mixed, active, thermic Typic Endoaqualfs) during 2019 and 2020. The soil in the experimental field was characterized by 21.54% sand, 57.62% silt, and 21.04% clay (Table 1). Soil bulk density averaged over 60‐cm depth was 1.36 g cm^−3^, and field‐saturated hydraulic conductivity (K_fs_) measured with a Saturo Infiltrometer (Meter Group Inc., WA, USA) ranged between 0.36 and 0.59 cm h^−1^. Field preparation consisted of one or two deep tillage events to break clay pans and overturn soils, burying crop residue, and killing weeds, followed by a disc‐tillage to generate furrows and ridges (102‐cm row spacing) and to facilitate furrow irrigations, conducted in the fall season after harvesting the previous crop. The raised‐ridge seedbeds were re‐furbished during the spring season, and the tops of the seedbeds were flattened before planting with Almaco cone plot planter (Allen Machine Company, Nevada, IA, USA). Each plot consisted of 4 rows spaced 102‐cm apart and 10‐m long. Seeding depth was adjusted to place the seed approximately 2.5‐cm deep in the soil. The planter was set to achieve an overall plant population density of approximately 336,000 plants ha^−1^. The Mississippi State University recommends a seeding rate of 345,800 seeds ha^−1^ for an MG IV soybean planted in April to May on clay soil (Smith et al. 2019). Plots were maintained weed‐free using both pre‐emergence and post‐emergence herbicide programs. No fertilizer was applied, and insect control programs were standard for soybean production.

**TABLE 1:** Selected soil (Dundee silt loam) physical and chemical properties of the experimental site in Stoneville, MS. Soil samples were collected and analyzed before planting in 2019 and 2020.

Crop season	Soil depth, cm	Soil texture	pH	Organic matter, %	CEC Meq 100 g^−1^	Mehlich‐3 extractable nutrients mg Kg^−1^						
						P	K	Ca	Mg	Zn	S	Cu
2019	0–15	Clay	7.22	1.88	24.6	68	288	6677	1245	2.5	9.9	3.8
	15–30	Clay	6.98	1.85	26.6	49	406	7185	1221	2.9	11.3	5.4
	30–45	Clay	6.88	1.47	25.3	28	242	4122	669	2.2	20.1	4.2
2020	0–15	Clay	7.12	1.96	24.8	55	384	1636	1155	2.4	9.5	4.0
	15–30	Clay	6.93	1.83	25.8	44	252	4243	938	2.5	6.8	5.2
	30–45	Clay	6.85	1.52	25.7	30	241	3865	902	2.0	18.6	3.9

Two cultivars belonging to MG III (cv. P37A78 and LG03‐4561‐14), three cultivars of MG IV (cv. Dyna‐gro 4516×, DS 25‐1, and DT97‐4290), and two cultivars of MG V (cv. S12‐1362 and S14‐16306) were used in the study. The field experiment was arranged as a split‐plot design, with irrigation (irrigated and rainfed) as the main plot factor and soybean cultivar as the subplot factor, replicated across four blocks (Table 2). Soybeans were planted on April 30, 2019 and May 2, 2020. Sensors for measuring soil‐matrix water potential (Irrometer, Riverside, CA, USA) were installed after calibration (Berrada et al. 2001) at depths of 15, 30, and 45 cm in selected representative plots that is, three sensors in each of the MG III, IV and V cultivars, and irrigations were applied based on a soil matrix potential of about −90 kPa at 45‐cm recommended soil depths (Plumblee et al. 2019). Flow meters (Mc Propeller flow meter, McCrometer, Hemet, CA, USA) were used for measuring applied irrigations. In 2019, 7.3 cm of water was applied in the irrigated plots in two events by applying water through the furrows on June 28th and July 29th. Total irrigation applied in 2020 was 9.8 cm, in two irrigation events of 4.9 cm each on June 18 and August 4th. The crop was not irrigated after the pod development stage (R6). Weather observations used in this study were collected by the Mid‐South Agricultural Weather Service, Delta Research and Extension Center, Stoneville, MS, which is located within a mile from the experimental field. The SY was assessed by harvesting the center two rows of each plot using a combined weighing system and adjusted for 13% moisture.

**TABLE 2:** Key characteristics of the soybean cultivars and their maturity groups used in the study.

S. No.	Maturity group	Genotype	Important trait(s)	Source
1	III	LG03‐4561‐14	High temperature stress tolerance	USDA‐ARS
2		P37A78	Roundup ready hybrid	Pioneer
3	IV	DT97‐4290	Charcoal rot tolerance	USDA‐ARS
4		DS 25‐1	Drought tolerance	USDA‐ARS
5		Dyna‐gro4516×	Popular cultivar in MS Delta	Loveland Inc. (Dyna Gro seed)
6	V	S14‐16306^a^	Flood tolerance	University of Missouri
7		S12‐1362^a^	Flood tolerance	University of Missouri

^a^ Not released.

### 2.1 Gas Exchange and Chlorophyll Fluorescence Measurements

At the beginning seed fill (R5 stage), physiological traits were analyzed using an LI‐6800 portable photosynthetic system (Li‐Cor Inc., Lincoln, Nebraska, USA). R5 was chosen as it is the most critical stage for soil moisture stress, which can lead to significant yield penalties (Cui et al. 2019; Eck et al. 1987). Measurements were made between 9:00 and 11:30 AM on five randomly selected plants from each plot on sunny cloud‐free days and wind velocity lower than 10 km hr.^−1^ as higher windspeed reduces stomatal conductance (El‐Sharkawy 1990). The third fully developed leaf from the apex of the plant was used, which is considered diagnostic for nutritional analysis, as the site of metabolic processes responsible for energy acquisition (Cotrim et al. 2021). The light source in the LI‐6800 was set at 1300 μmol (photon) m^−2^ s^−1^ (90% red and 10% blue light) to measure the net photosynthetic rate. The leaf temperature was kept at 31°C ± 1.8°C, the CO_2_ concentration was maintained at 400 μmol mol^−1^, relative humidity was 60%–65%, and airflow was 500 μ mol s^−1^. The photosynthetic traits measured were net photosynthetic rate (A, μmol CO_2_ m^−2^ s^−1^), transpiration rate (E, mmol H_2_O m^−2^ s^−1^), stomatal conductance (g_s_, mol H_2_O m^−2^ s^−1^), internal CO_2_ concentration (Ci, μmol CO_2_ mol^−1^), instantaneous water‐use efficiency (WUE) and instantaneous carboxylation efficiency (ICE). The WUE was calculated as the ratio between A and E (μmol CO_2_ per mmol H_2_O) and ICE was calculated as the ratio between A and Ci (μmol CO_2_ m^−2^ s^−1^ per μmol CO_2_ mol^−1^). The Chlorophyll fluorescence parameters were detected using an integrated fluorometer in LI 6800. The quantum yield of PSII photochemistry (ΦPSII) and photosynthetic electron transport (ETR) were measured using multiphase flash type, and the other parameters were set to default.

$$\text{ΦPSII}=\left({\mathrm{Fm}}^{\prime }-\mathrm{Fs}\right)/{\mathrm{Fm}}^{\prime }$$

where Fs is steady‐state fluorescence and Fm′ is maximal fluorescence under light‐adapted conditions.

### 2.2 Statistical Analysis

All statistical analyses were conducted using JMP Pro v. 14.1.0 (SAS Institute, Cary, NC, USA). To prevent pseudo replication, physiological measurements taken from the five individual plants within each subplot were first averaged to generate a single mean value per subplot for each measured variable (A, g_s_, Cᵢ, E, ETR, ΦPSII). These subplot means, along with the seed yield (SY) data, were then used for the subsequent analysis. The data were analyzed using a mixed linear model (PROC MIXED). The model was structured to correctly reflect the split‐plot design with repeated measures over years. The model statement was specified as follows:

- Fixed Effects: Year (Y), Irrigation (I), Cultivar (C), and all two‐ and three‐way interactions (Y × I, Y × C, I × C, Y × I × C).
- Random Effects: Replicate/Block (Rep), the main plot error term Rep × I(Y), and their interactions with year.

The appropriate error term for testing the main plot effect (Irrigation) and its interaction with Year was the *F*‐test using the mean square of Rep × I(Y) as the denominator. The subplot effects (Cultivar) and their interactions were tested against the residual error (mean square error, MSE). This structure ensures that the different error variances for the main plots and sub‐plots are correctly applied. Treatment means were separated using Tukey's Honestly Significant Difference (HSD) test at a significance level of *α* = 0.05. To understand the associations between physiological traits and final seed yield, a Pearson correlation matrix was computed using PROC CORR for each irrigation treatment separately, combining data from both years.

## 3 Results and Discussion

### 3.1 Weather

**FIGURE 1:**
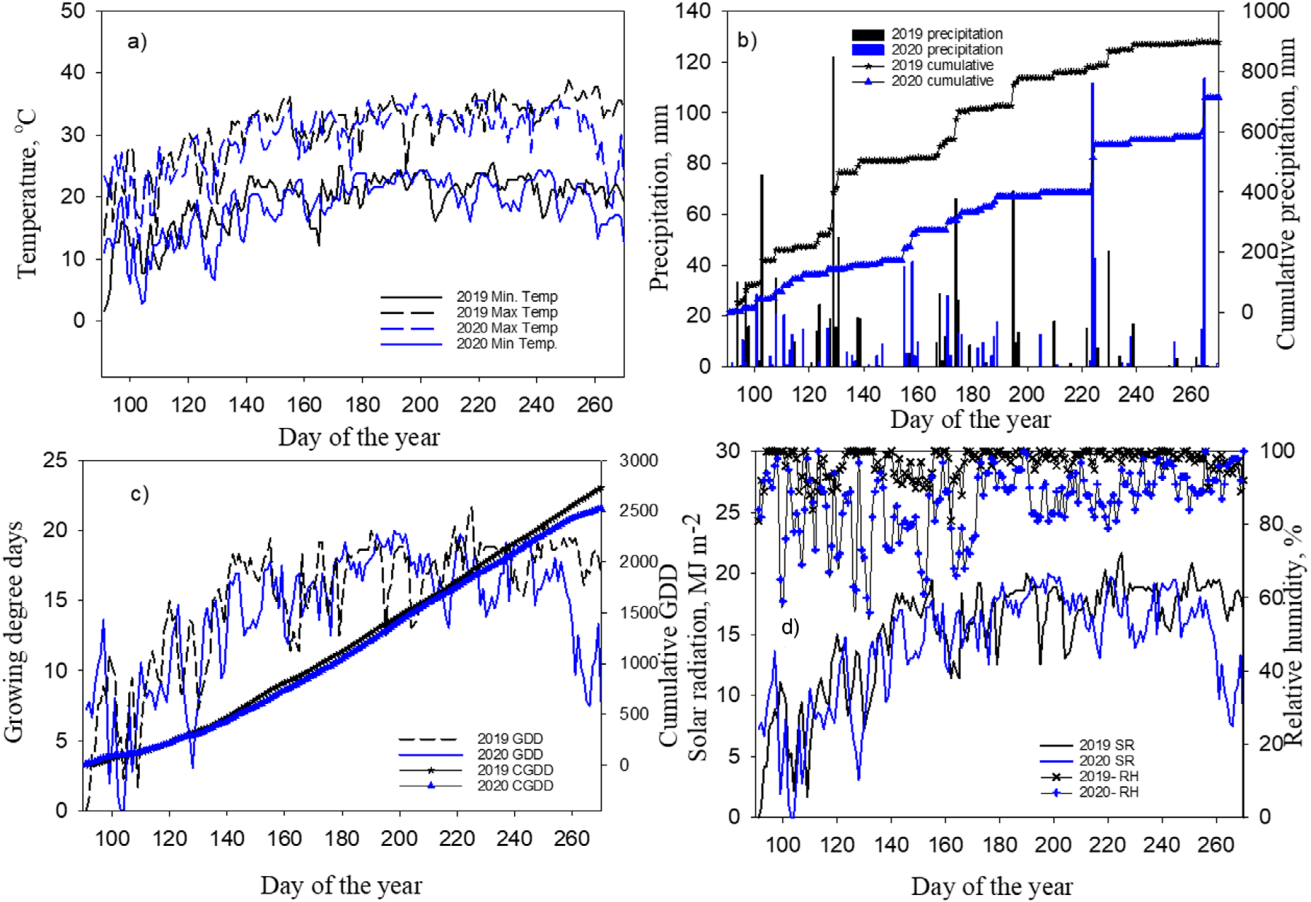
Measured (a) daily maximum (max.) and minimum (min.) air temperature, (b) daily and seasonal cumulative (cum.) precipitation, (c) daily (GDD) and seasonal cumulative (CGDD) growing degree days, and (d) daily solar radiation (SR) and relative humidity (RH) for 2019 and 2020 soybean growing seasons at Stoneville, MS, USA.

Contrasting weather patterns were observed during the 2019 and 2020 cropping seasons (Figure 1). The 2020 active soybean crop season (May–August) received 135% lower precipitation than in 2019 (272 vs. 638 mm). The number of rainy days during the peak reproductive period and pod maturation (June–August) in 2019 was 25, while in 2020 there were 29 rainy days. The soybean growth period (May–August) in 2019 had 41 GDD more than that of 2020. Similarly, averaged over May–August, the 2020 crop season recorded 26% higher daily maximum temperatures (32.2°C in 2020 vs. 25.4°C in 2019) and 47% higher daily minimum temperatures (21.48°C in 2020 vs. 14.4°C in 2019). These weather differences during the two crop seasons were reflected in cultivar performance for seed yield and related physiological traits as revealed in the analysis of variance (ANOVA) tests discussed below.

### 3.2 Grain Yield and Fluorescence Parameters and Parameters Related to Photosynthesis

The ANOVA revealed significant differences in seed yield (SY) and related photosynthetic and chlorophyll fluorescence traits such as A, Cᵢ, E, ICE, WUE, ΦPSII, and ETR among cultivars (Table 3). Cultivar and irrigation level had significant effects on SY, A, Cᵢ, E, ICE, WUE, ΦPSII, and ETR, while year affected all the traits except Cᵢ and WUE. Cultivar × irrigation level and Cultivar × year interactions had significant effects on A, E, Cᵢ, g_s_, ΦPSII, and ETR.

**TABLE 3:** Significance of the main effects of cultivar, irrigation level (IR and RF), year and their interactions.

	df	Seed yield	A (μmol CO_2_ m^−2^ s^−1^)	E (mmol m^−2^ s^−1^)	Ci (μmol mol^−1^)	WUE	ICE	g_s_ (mol H_2_O m^−2^ s^−1^)	ΦPSII	ETR
Cultivars	6	0.001*	< 0.001*	< 0.001*	< 0.001*	< 0.001*	< 0.001*	0.005*	< 0.001*	< 0.001*
Irrigation level	1	< 0.001*	0.0019*	0.002*	< 0.001*	< 0.001*	< 0.001*	< 0.002*	< 0.001*	0.001*
Year	1	0.004*	< 0.001	0.015*	0.104	0.059	0.002*	< 0.001*	0.0482*	0.025*
Cultivars × Irrigation level	6	0.976	< 0.001*	0.002*	< 0.001*	< 0.001*	0.0042*	0.611	0.007*	0.822
Cultivars × Year	6	0.0519	0.0038*	0.086	0.915	0.10	0.247	0.002*	0.021	0.003*
Irrigation level × Year	1	0.001*	< 0.001*	< 0.001*	0.1421	< 0.001*	0.0874	0.126	0.961	0.716
Cultivar × Irrigation level × Year	6	0.001*	0.621	0.11	0.10001	0.354	0.172	0.514	0.286	0.254

Abbreviations: ΦPSII, effective quantum yield of photosystem II; A, net photosynthetic rate; Ci, internal CO_2_ concentration; E, transpiration rate; ETR, electron transport rate; g_s_, stomatal conductance; ICE, instantaneous carboxylation efficiency; IR, irrigated; RF, rainfed; WUE, instantaneous water‐use efficiency.

* Significantly different at *p* ≤ 0.05 level.

**FIGURE 2:**
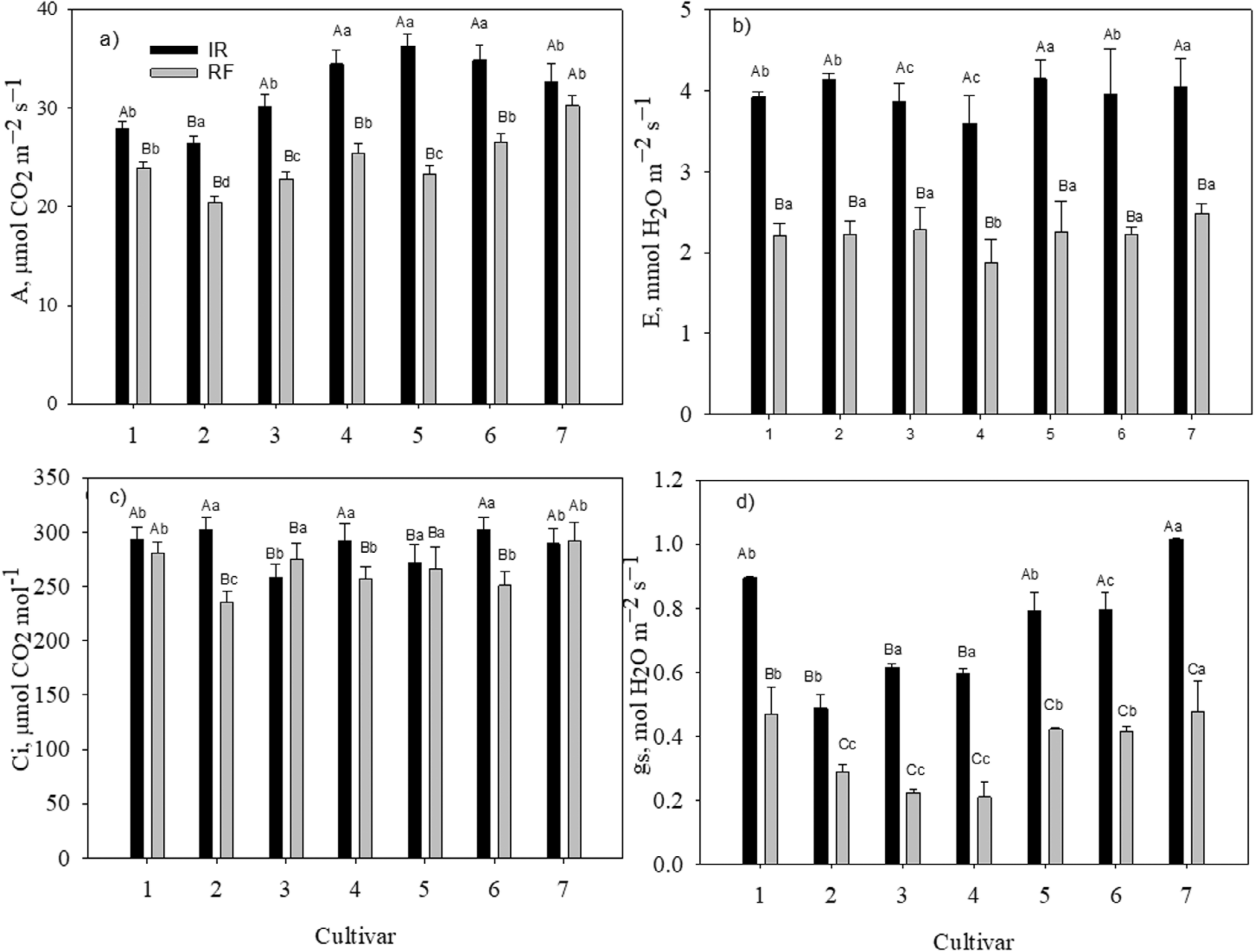
Effect of irrigation (IR) and rainfed (RF) systems on (a) net photosynthetic rate (A), (b) transpiration rate (E), (c) internal CO_2_ concentration (Ci) and, (d) stomatal conductance (g_s_), in seven soybean cultivars: (1) S14‐16306, (2) S12‐1362, (3) DT97‐4290, (4) DS 25‐1, (5) Dyna‐gro 4516×, (6) LG03‐4561‐14 and, (7) P37A78. Data show the means ± one standard deviation across replicates. Different letters on vertical bars indicate significant differences between means at the *p* < 0.05 level.

Photosynthesis is a good indicator of plant growth and metabolism owing to its significant association with biomass production and SY (Ashraf and Harris 2013). The highest A (36.7 μmol CO_2_ m^−2^ s^−1^) was recorded by DS 25–1 under irrigated conditions, with a range between 26.4 to 36.7 μmol CO_2_ m^−2^ s^−1^; under RF, it varied between 20.4 to 30.3 μmol CO_2_ m^−2^ s^−1^ (Figure 2a). The impact of irrigation on A was highest in the cultivar Dyna‐gro 4516× (55% higher), followed by DS 25‐1 and LG03‐4561‐14. Similar results of significantly higher A for irrigated soybeans were earlier reported by several researchers (Gilbert et al. 2011). Another study showed that moisture deficit stress reduces stomatal conductance, lowering the A rate, but it was also genotype‐dependent (Tong et al. 2019).

The intercellular CO_2_ concentration (Cᵢ) is a good indicator of A. Unlike other traits, the variability for Cᵢ across IR and RF conditions was not large. Cᵢ ranged between 257 and 302 μmol CO_2_ mol^−1^ of air in the IR system while varying from 235 to 292 μmol CO_2_ mol^−1^ in the RF system (Figure 2c). The cultivar S12‐1362 recorded the highest Cᵢ of 302.7 μmol mol^−1^ under IR, while MG III cultivar P37A78 recorded the highest Cᵢ of 292 μmol mol^−1^ under moisture deficit conditions. It appears that the difference in the Cᵢ response to irrigation among cultivars is genotype‐driven rather than determined by the moisture regime; however, this observation requires further research to substantiate.

Further, in the absence of other limiting factors, higher Cᵢ had a very significant positive association with A, while many instances of lower A are coupled with lower Cᵢ in the presence of both biotic and abiotic stresses (Urban et al. 2017). The stomatal conductance (g_s_) varied between 0.49 and 1.01 mol H_2_O m^−2^ s^−1^ in the IR crop, while a significantly lower and narrower range was observed under moisture deficit conditions (0.21–0.48 mol H_2_O m^−2^ s^−1^). The lowest g_s_ of 0.21 was observed in DS25‐1, a released drought‐tolerant line from the USDA‐ARS. The drought‐tolerant mechanism of DS25‐1 appears to involve saving water by significantly reducing g_s_ while impacting A less (Figure 2a,d). Several reports showed that stomatal conductance drops drastically with drought stress, resulting in increased WUE (net CO_2_ assimilation rate/transpiration rate) (Cotrim et al. 2021; Gilbert et al. 2011; Jumrani and Bhatia 2019; Urban et al. 2017). With mild water stress, reduced stomatal conductance is known to have a larger inhibitory effect on E than on CO_2_ diffusion into the leaf tissues (Ohashi et al. 2006). However, under severe stress, the decreased leaf turgor and air vapor pressure deficit (VPD), besides root‐generated chemical signals, impact mesophyll conductance, leading to lower Cᵢ and WUE (Gilbert et al. 2011; Jumrani and Bhatia 2019; Urban et al. 2017).

**FIGURE 3:**
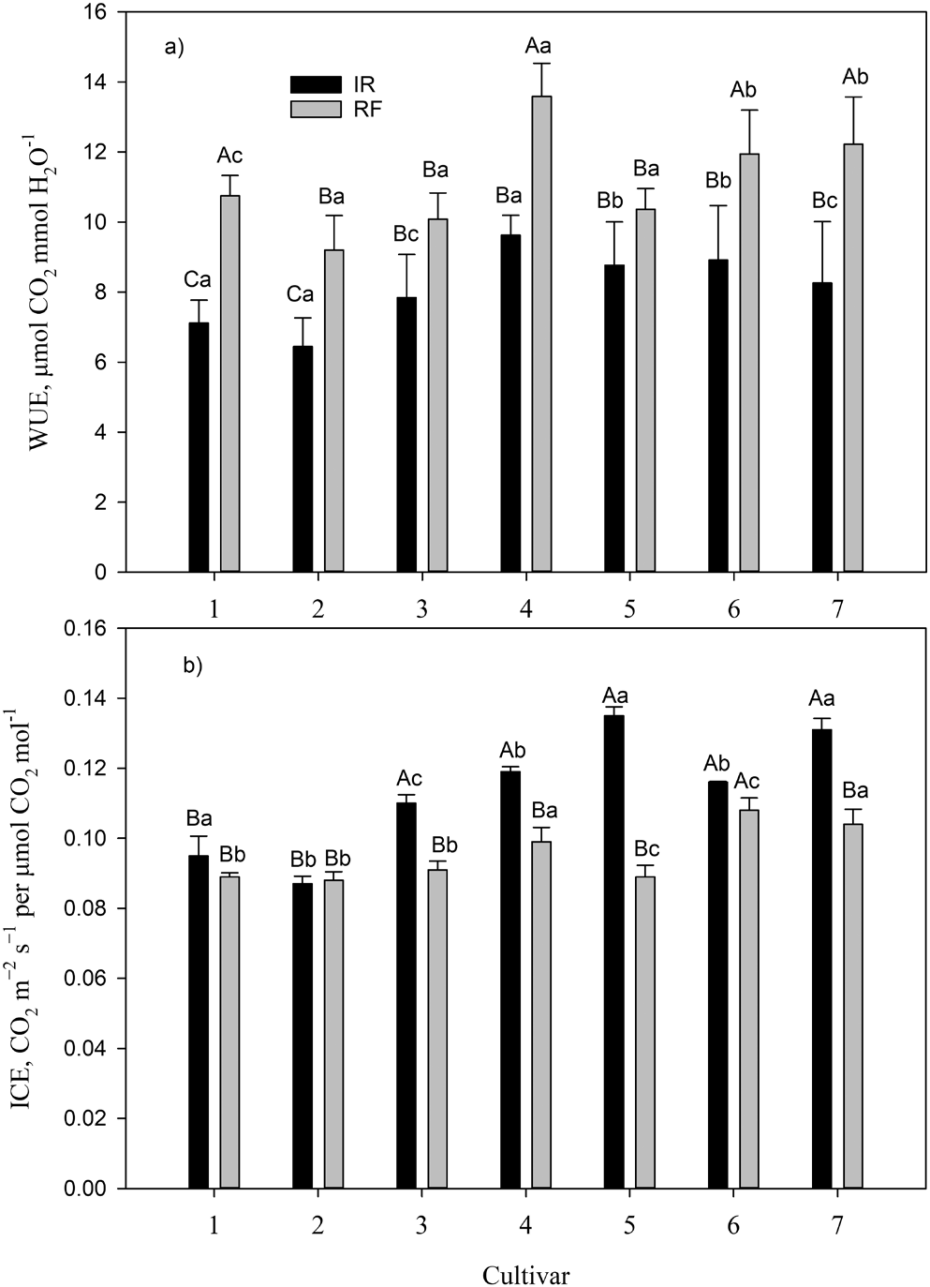
Effect of irrigation (IR) and rainfed (RF) systems on (a) instantaneous water use efficiency (WUE), and (b) instantaneous carboxylation efficiency (ICE), in seven soybean cultivars: (1) S14‐16306, (2) S12‐1362, (3) DT97‐4290, (4) DS25‐1, (5) Dyna‐gro 4516×, (6) LG03‐4561‐14 and (7) P37A78. Data show the means ± standard deviation of replicates. Different letters on vertical bars indicate significant differences between means at the *p* < 0.05 level.

The instantaneous water‐use efficiency (WUE) is being used to screen for heritable genotypic variation in water use parameters of multiple crops (Jumrani and Bhatia 2019). The grouping of means for WUE under IR and RF is presented in Figure 3a. The WUE was significantly higher in the RF crop by 18%–51%, depending on the cultivar. WUE ranged from 6.4 to 9.6 μmol CO_2_ per mmol H_2_O under IR, while it ranged from 9.2 and 13.6 μmol CO_2_ per mmol H_2_O under RF. The cultivar DS25‐1 had the highest WUE in both IR and RF conditions (9.6 in IR and 13.6 μmol CO_2_ per mmol H_2_O). As WUE is the ratio of A to E, higher E will automatically result in lower WUE due to their inverse relationship. The current results echo the findings of previous reports on soybean responses to abiotic stresses (Cotrim et al. 2021; El‐Sharkawy 1990; Gilbert et al. 2011; Jumrani and Bhatia 2019). In field conditions, variation in E and g_s_ will probably have a larger impact on WUE than A due to the strong association of g_s_ with A than the association of E with A (Gilbert et al. 2011; Pinnamaneni et al. 2021). Hence, cultivars with higher g_s_ and A rates and lower E rates could be viable for breeding soybean cultivars for mild and severe moisture deficit conditions.

The performance of cultivars for instantaneous carboxylation efficiency (ICE) is shown in Figure 3b. It ranged from 0.09 to 0.14 μmol CO_2_ m^−2^ s^−1^ per μmol CO_2_ mol^−1^ in the IR crop, while the range for the RF crop was from 0.09 to 0.11 μmol CO_2_ m^−2^ s^−1^ per μmol CO_2_ mol^−1^. The cultivar Dyna‐gro 4516× had the highest ICE of 0.14 in IR, while LG03‐4561‐14 recorded the highest ICE in the RF system (0.11). Unlike the clear trend established for WUE, there appears to be a confounding effect of g_s_, E, and A on ICE, as vindicated by the non‐significant effect of irrigation on ICE for S14‐16306, S12‐1362, and LG03‐4561‐14. The current study results conform to the observations of earlier published reports (Cotrim et al. 2021; Jumrani and Bhatia 2019).

**FIGURE 4:**
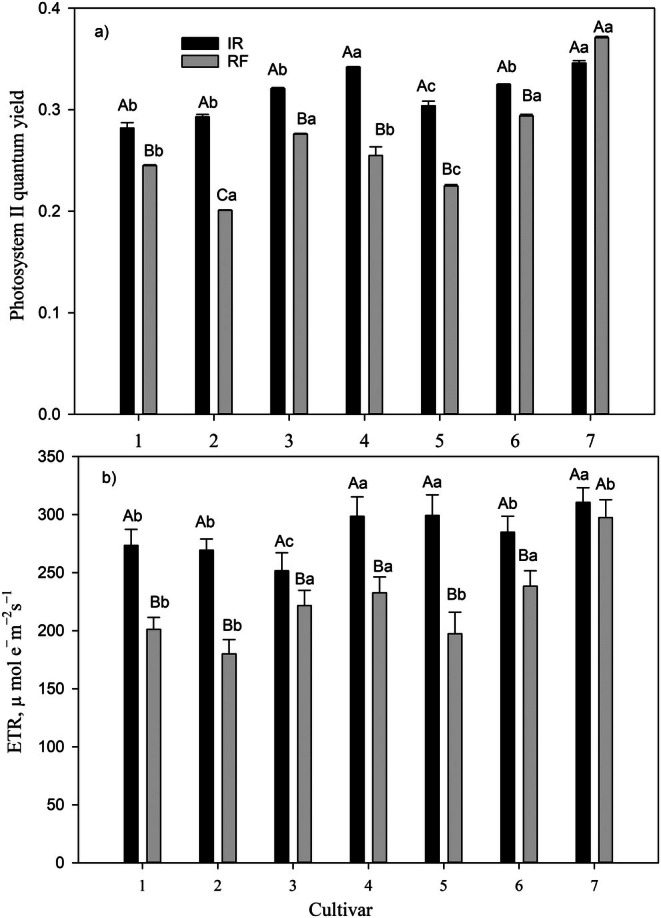
Effect of irrigation (IR) and rainfed systems (RF) on (a) photosystem quantum yield (ΦPSII) and (b) electron transport rate (ETR) in seven soybean cultivars: (1) S14‐16306, (2) S12‐1362, (3) DT97‐4290, (4) DS 25‐1, (5) Dyna‐gro 4516×, (6) LG03‐4561‐14 and (7) P37A78. Data show the means ± standard deviation of replicates. Different letters on vertical bars indicate significant differences between means at the *p* < 0.05 level.

We have estimated two parameters of Chlorophyll fluorescence: the quantum yield of photosystem II (ΦPSII) and the electron transport rate (ETR) (Figure 4a,b). ΦPSII was significantly higher in all cultivars under IR except for P37A78. ΦPSII ranged from 0.28 to 0.35 under IR conditions, while for RF crops it varied between 0.20 and 0.37. The cultivar S12‐1362 recorded a significant impact of irrigation on ΦPSII with an increase of 45% over RF conditions. The drought‐tolerant cultivar DS 25‐1 recorded a 34% higher ΦPSII (IR = 0.34 and RF = 0.25). The positive effect of irrigation on ΦPSII was earlier reported (Urban et al. 2017). The ETR was significantly higher in all irrigated cultivars except for P37A78 (Figure 4b). This data corroborates the earlier observation for ΦPSII. Across the cultivars evaluated, irrigation impacted ETR positively to the extent of 13% to 52%. The highest impact of irrigation on ETR was observed for cultivar Dyna‐gro 4516×.

The data on seed yield per plant and 100‐seed weight of the evaluated cultivars are given in Table 4. In the 2019 season, among the MG IV cultivars, Dyna‐gro 4516× recorded the highest per‐plant seed yield of 23.73 and 20.16 g under IR and RF conditions, respectively. MG V cultivars LG03‐4561‐14 and DS 25‐1 had per‐plant yields of 23.26 and 20.52 g under IR, and 20.36 and 21.09 g in RF conditions, respectively. In the 2020 season, cultivars Dyna‐gro 4516×, LG03‐4561‐14, and DS 25‐1 recorded per‐plant yields of 29.08, 23.37, and 23.12 g under IR, and 25.59, 21.68, and 21.04 g under RF, respectively. The higher seed yield of these lines was accompanied by significantly higher A, ICE, WUE, ETR, and g_s_ under both IR and RF conditions (Figures 2, 3, 4), suggesting a physiological association between these traits and productivity, though causation cannot be inferred from correlative data alone. MGIV cultivars DS 25‐1 and LG03‐4561‐14 appear well suited to both IR and RF production systems in the Mississippi Delta.

**TABLE 4:** Seed yield productivity of different maturity group cultivars under irrigated (IR) and rainfed (RF) conditions during 2019–20 in Stoneville, Mississippi, USA.

Genotype	Irrigation level	Seed yield (g plant^−1^)		100‐seed weight (g)	
		2019	2020	2019	2020
S14‐16306 (MG V)	IR	14.36 d	16.11d	17.1 c	17.0 d
	RF	13.68 d	13.60 e	16.9 d	16.7 d
S12‐1362 (MG V)	IR	18.02 bc	16.48 d	18.9 c	17.5 d
	RF	18.21 bc	13.91 e	16.1 d	15.9 e
DT97‐4290 (MG III)	IR	19.85 b	19.63 cd	19.1 b	18.7 c
	RF	17.49 c	15.76 d	18.4 c	18.2 c
DS 25‐1 (MG IV)	IR	20.52 b	23.12 a	18.9 c	18.8 c
	RF	21.09 b	21.04 c	18.6 c	18.3 c
Dyna‐gro 4516× (MG III)	IR	23.73 a	29.08 a	20.9 a	20.8 a
	RF	20.16 b	25.59 ab	20.7 a	20.4 a
LG03‐4561‐14 (MG IV)	IR	23.26 a	23.37 bc	19.7 b	19.8 b
	RF	20.36 b	21.68 bc	19.5 b	19.3 b
P37A78 (MG III)	IR	19.95 b	20.55 c	20.8 a	20.9 a
	RF	19.74 b	19.75 c	20.1 b	19.9 b

*Note:* Different letters indicate significant differences between means at the *p* < 0.05 level.

### 3.3 Correlation Among Traits

The Pearson correlation coefficients among the photosynthetic traits and SY under IR conditions are presented in Table 5. A and ICE are significantly correlated with SY (*r* = 0.76 and 0.78, respectively). These results echo the findings of earlier reports on wheat (Vilfan et al. 2019) and soybean (Cotrim et al. 2021), who found a strong correlation of A and ICE with SY. Other traits such as WUE, E, and ΦPSII had a significant positive correlation with SY. A has a strong correlation with all the traits studied except for ETR. It is not surprising to note the highly significant negative correlation of Cᵢ with ICE, as enhanced CO_2_ assimilation reduces the internal concentration of CO_2_. ICE is a measure of CO_2_ assimilation rate, which is generally high when plants are not subjected to limiting factors for A, like photosynthetically active radiation, CO_2_ concentration, air temperature, and, most importantly, soil moisture.

**TABLE 5:** Pearson correlation between the photosynthetic and chlorophyll fluorescence related traits evaluated in irrigated (IR) soybean for the seven soybean cultivars: Net photosynthetic rate (A), stomatal conductance (g_s_), internal CO_2_ concentration (Ci), transpiration rate (E), instantaneous water‐use efficiency (WUE), instantaneous carboxylation efficiency (ICE), photosystem 2 quantum yield (ΦPSII), electron transport rate (ETR) and seed yield (SY). The experiment was conducted in Stoneville, MS, USA, during 2019–2020. Italicized coefficients are for rainfed (RF) while normal font coefficients are for IR.

	SY	A	E	Ci	WUE	ICE	g_s_	ΦPSII	ETR
SY	1.00	*0.53* *	*−0.20* *	ns	*0.73* **	*0.46* *	*−0.37* *	*0.65* **	*0.67* **
A	0.76**	1.00	*0.27* *	ns	*0.80* ***	*0.88* **	ns	*0.71* **	*0.88* ***
E	0.39*	0.37*	1.00	ns	*−0.36* *	ns	*0.74* *	*0.33* *	ns
Ci	−0.55*	−0.52*	ns	1.00	ns	*−0.47* *	ns	*0.49* *	ns
WUE	0.37*	0.62*	−0.49*	−0.35*	1.00	*0.77* **	ns	*0.45* *	*0.73* **
ICE	0.78**	0.91***	0.33*	−0.93***	0.56*	1.00	ns	*0.40* *	*0.66* **
g_s_	ns	0.36*	0.71**	ns	ns	ns	1.00	ns	ns
ΦPSII	0.42*	0.61*	ns	−0.45*	0.62*	0.59*	ns	1.00	*0.81* *
ETR	ns	0.42*	0.30*	ns	ns	*0.34* *	*0.31* *	*0.34* *	*1.00*

Abbreviation: ns, not significant.

* Significance at *p* ≤ 0.05.

** Significance at *p* ≤ 0.01.

*** Significance at *p* ≤ 0.001.

Under the RF regime, SY has a strong positive correlation with A, WUE, ΦPSII, and ETR (*r* = 0.53, 0.73, 0.67, and 0.65, respectively) (Table 5). Compared with the IR regime, the association of A with SY is lower (*r* = 0.76 in IR and 0.53 in RF). Other parameters such as WUE, ΦPSII, and ETR play a greater role in determining SY under the RF regime. Both E and g_s_ are negatively correlated with SY (*r* = −0.37 and −0.20, respectively). These observations differ from the earlier reports on SY correlation with g_s_ and WUE (Cotrim et al. 2021; Stinziano et al. 2019) but conform with the association of A with SY. This could be due to genetic differences in the cultivars, growing conditions, and phenological differences at the time of measurements. The current study was performed at the R5 stage for all the cultivars, while another study collected data 60 days after emergence uniformly across genotypes without considering the variability in days to maturity (Cotrim et al. 2021). It may be noted that ICE, WUE, ΦPSII, and ETR had a strong correlation with photosynthesis, echoing earlier observations (Gilbert et al. 2011; Jumrani and Bhatia 2019). These findings suggest that under RF conditions, WUE, ΦPSII, and ETR may serve as complementary selection indicators alongside assimilation rate, although this inference is drawn from a relatively small set of cultivars at a single location and requires further validation.

High g_s_ rates are critical to the optimum growth and yield of modern genetically engineered crops, particularly during seed formation and filling (Roche 2015). It was further noted that higher rates of g_s_ are needed for maintaining the soil–plant‐atmosphere continuum. This study highlights the association of WUE with SY rather than the association of g_s_ with SY, probably due to the genetic constitution of the cultivars. Only cv. Dyna‐gro 4516× and P37A78 are transgenic, while the remaining cultivars were traditionally bred. These observations indicate that A and WUE, along with ΦPSII and ETR, were associated with favorable yield outcomes under both water regimes in this study, pointing to their potential utility as physiological markers in trait‐based selection. However, the correlations observed here do not establish causality, and the relative importance of these parameters likely varies with the degree and timing of moisture deficit, as well as genetic background. Multi‐environment trials with larger and more diverse germplasm panels are needed to confirm whether these trait–yield associations are robust enough to inform breeding decisions.

### 3.4 WUE and SY

The WUE was a critical parameter limiting yield gains in rice (Zheng et al. 2011) and soybean (Liu et al. 2012). Intrinsic water use efficiency (WUE), defined as the ratio of net photosynthetic rate to stomatal conductance for water vapor (A/g_s_), plays an increasingly important role in sustaining grain productivity under limited soil moisture conditions (Note: this is distinct from the instantaneous WUE, A/E, discussed in Section 3.2). In this study, WUE (A/E) showed a significant correlation (*r* = 0.73) with seed yield under rainfed conditions, consistent with previous reports (Liu et al. 2012; Zheng et al. 2011), likely due to stomatal kinetics and the associated mitigation of intercellular CO_2_ depletion. In drier soils, poor soil hydraulic conductivity severely limits water uptake, depending on soil texture, leading to the closure of stomata (Abdalla et al. 2021; Carminati et al. 2020; Pelech et al. 2021). But further time‐series studies are required to delineate the underlying mechanisms, subject to the differences in phenology of the maturity groups. Both ΦPSII and ETR are impacted by soil moisture stress based on the extent of the stress (Jumrani and Bhatia 2019; Roche 2015; Tong et al. 2019; Vongcharoen et al. 2019). In this study, both ΦPSII and ETR exhibited a strong correlation to both SY and A, conforming with previous reports (Jumrani and Bhatia 2019; Roche 2015; Tong et al. 2019; Zheng et al. 2011). PSII is not only involved in light harvesting but also in the photolysis of water and ETR; hence, any reduction in PSII could negatively impact crop productivity.

## 4 Conclusions

This study investigated the relationship between photosynthetic traits and seed yield (SY) in seven soybean cultivars under IR and RF conditions in the Mississippi Delta. We identified distinct physiological correlates of yield that were contingent on water availability. Under irrigated conditions, A and ICE were most strongly associated with SY. In contrast, under the water‐limited RF regime, WUE, ETR, and ΦPSII emerged as the key traits linked to higher SY. The following cultivars, notably DS 25‐1, Dyna‐Gro 4516×, and LG03‐4561‐14 (MG IV‐V), and P37A78 (MG III), demonstrated superior photosynthetic performance and correspondingly higher yields across both water regimes. This suggests a potential link between these physiological markers and yield stability. However, these findings must be interpreted with caution. As this study was limited to seven cultivars at a single location over two seasons, the results highlight promising associations rather than establishing causal relationships or providing definitive breeding directives. The correlations observed should be viewed as preliminary evidence that traits like WUE and ETR could serve as valuable complements to traditional selection criteria. Future research should aim to validate these trait‐yield relationships across more diverse genetic backgrounds and environments. Confirming the heritability and consistent expression of these traits is a critical next step. If these associations prove robust, the integration of these photosynthetic parameters into breeding pipelines could enhance selection efficiency, contributing to the development of soybean cultivars with improved performance and resilience in both irrigated and rainfed production systems. This work provides a foundational comparison of photosynthetic parameters across maturity groups III–V within the ESPS, offering a starting point for this broader research effort.

## Funding

This research was funded by USDA‐ARS under the project number: 6066‐22000‐089‐000D and 6054‐41000‐114‐000‐D.

## Disclosure

The use of trade, firm, or corporation names in this paper is for the information and convenience of the reader. Such use does not constitute an official endorsement of approval by the United States Department of Agriculture (USDA) of any product or service to the exclusion of others that may be suitable.

## Consent

All Authors have provided consent for publication and ethical approval.

## Conflicts of Interest

The authors declare no conflicts of interest.

## Data Availability

The data that supports the findings of this study were available in the manuscript file.
